# Molecular detection and genetic characterization of *Giardia duodenalis* in temple stray cats in Bangkok, Thailand

**DOI:** 10.3389/fpara.2026.1948035

**Published:** 2026-09-15

**Authors:** Vy Thi Tuong Le, Pornkamol Phoosangwalthong, Minh-Trang Thi Hoang, Tawin Inpankaew

**Affiliations:** 1 Department of Parasitology, Faculty of Veterinary Medicine, Kasetsart University, Bangkok, Thailand; 2 Department of Microbiology and Parasitology, Buon Ma Thuot Medical University, Buon Ma Thuot, Dak Lak, Vietnam

**Keywords:** associated factors, feline giardiasis, molecular epidemiology, phylogenetic analysis, zoonotic assemblage

## Abstract

**Introduction:**

*Giardia duodenalis* is a ubiquitous enteric protozoan infecting humans, domestic animals, and wildlife worldwide. In Thailand, high densities of stray animals represent an important management challenge. However, molecular epidemiological data regarding stray cats remain limited. This study estimated the prevalence of *G. duodenalis*, characterized the identified genetic assemblages, and explored factors associated with PCR positivity in temple stray cats in Bangkok, Thailand.

**Methods:**

Fecal samples were collected from 500 stray cats residing across 43 temples in 24 districts of Bangkok. Detection of *G. duodenalis* was performed using nested PCR targeting the small subunit ribosomal RNA (SSU rRNA) gene. Positive samples were further characterized by genotyping at the glutamate dehydrogenase (*gdh*) and triosephosphate isomerase (*tpi*) loci. Phylogenetic analyses were conducted using the Maximum Likelihood method based on the Tamura 3-parameter model. Associated factors were evaluated using multivariable logistic regression.

**Results:**

The overall prevalence of *G. duodenalis* was 10.8% (54/500; 95% CI: 8.4–13.8%), with positive cases identified in 12 of the 24 surveyed districts and 19 of the 43 sampled temples. Eight isolates were successfully genotyped, identifying assemblage B (sub-assemblage BIV, n = 4), assemblage F (n = 3), and assemblage A (sub-assemblage AI, n = 1). The absence of human contact was identified as an exploratory variable associated with higher odds of PCR positivity (adjusted odds ratio [aOR] = 8.80; 95% CI: 2.15–35.98; *P* = 0.002), while demographic factors (age, sex), geographic zone, and deworming history showed no statistically significant associations.

**Discussion:**

Assemblages previously reported in humans and various animals were detected among a small number of successfully genotyped isolates, while the source, direction of transmission, and the reservoir status of temple stray cats could not be determined.

## 1 Introduction

Giardiasis is a widespread intestinal parasitic infection of significant veterinary and public health concern, recognized as one of the most common waterborne and foodborne diseases (Jerez Puebla et al., 2017; Sun et al., 2023). It is estimated that 200 million people are infected globally (Certad et al., 2017). Giardiasis transmission can occur through the fecal-oral route, either directly via contact with feces from infected humans or animals, or indirectly through the consumption of food and water contaminated with infective cysts (Ballweber et al., 2010; Dixon, 2021). Clinical manifestations range from asymptomatic to acute or chronic infection, with common symptoms including diarrhea, weight loss, abdominal pain, and malabsorption (Jerez Puebla et al., 2017; Kurnosova et al., 2024).

*G. duodenalis* is the only species within the genus *Giardia* known to infect both humans and a wide variety of domesticated and wild mammals (Guadaño Procesi et al., 2022; Sprong et al., 2009). Despite morphological similarities, molecular genotyping has classified the species into eight distinct genetic assemblages (A-H) (Kraft et al., 2017; Rahimi et al., 2023; Zajaczkowski et al., 2021). Assemblages A and B are considered zoonotic, infecting a broad range of hosts including humans and various animals (Cai et al., 2021; Tangtrongsup et al., 2020), whereas assemblages C–H exhibit host specificity. Assemblages C and D primarily infect canids, assemblage E is found in domestic and wild ungulates, assemblage F mainly infects felines, and assemblages G and H are commonly found in rodents and seals, respectively (Ryan et al., 2021).

Sub-assemblages have been further characterized within the zoonotic assemblages A (AI, AII, and AIII) and B (BIII and BIV) (Feng and Xiao, 2011; Piekara-Stępińska et al., 2020; Ryan and Cacciò, 2013). Sub-assemblage AI is considered zoonotic (Faria et al., 2017; Hernández et al., 2019), and AII mainly infects humans but retains zoonotic potential (Ryan and Cacciò, 2013), while AIII is primarily restricted to wild hoofed animals (Cacciò et al., 2008; Feng and Xiao, 2011; Iwashita et al., 2020). Sub-assemblages BIII and BIV are commonly found in humans (Zajaczkowski et al., 2021) and have also been reported in various animal hosts (Sprong et al., 2009). The broad host range of *G. duodenalis* increases the risk of cross-species transmission, making human giardiasis a continuing public health concern (Hatam-Nahavandi et al., 2025).

Companion animals may play a role in the zoonotic transmission dynamics of giardiasis (Sun et al., 2023). Globally, *G. duodenalis* infection in cats has an estimated pooled prevalence of 14.5% (Sun et al., 2023). Notably, cats can harbor not only the feline-adapted assemblage F but also the zoonotic assemblages A and B, and occasionally canine-adapted assemblages C and D (Li et al., 2024; Saleh et al., 2019; Yun et al., 2023). Thailand has a large population of abandoned and stray cats (Phoosangwalthong et al., 2023). They often lack veterinary attention and are exposed to extensive environmental contamination, increasing their susceptibility to parasitic infections (Phoosangwalthong et al., 2022). These stray cats frequently reside in public areas, particularly temples, facilitating contact with humans and other animals. This close contact with humans and shared environment make this population highly relevant for molecular epidemiological investigation.

In Thailand, *G. duodenalis* has been reported in humans (Kitvatanachai et al., 2021; Pipatsatitpong et al., 2018; Siripattanapipong et al., 2011; Tungtrongchitr et al., 2010), livestock (Inpankaew et al., 2015; Khurajog et al., 2014), wildlife (Sricharern et al., 2016; Tangtrongsup et al., 2019), companion animals, including dogs (Inpankaew et al., 2007; Khine et al., 2021; Rojekittikhun et al., 2014; Tangtrongsup et al., 2017, 2020), and cats (Jitsamai et al., 2021; Rojekittikhun et al., 2014; Tangtrongsup et al., 2020), as well as exotic pets (Kamyingkird et al., 2022). Despite these reports, epidemiological information on *G. duodenalis* infection in cats in Thailand remains limited. To date, few studies have investigated *G. duodenalis* infection in owned and sheltered cats in Thailand, while stray cat populations remain uninvestigated. Among the existing studies, only one performed molecular characterization (Tangtrongsup et al., 2020), whereas the remaining studies relied solely on microscopic examination or coproantigen detection without genotyping (Jitsamai et al., 2021; Rojekittikhun et al., 2014).

The present study aimed to determine the prevalence of *G. duodenalis* in temple stray cats in Bangkok, Thailand, characterize the identified genetic assemblages using molecular methods, and explore factors associated with PCR positivity.

## 2 Materials and methods

### 2.1 Study area and sample collection

A cross-sectional study was conducted from June to December 2015 among temple stray cats in Bangkok, Thailand. Temple stray cats were defined as unowned cats residing within the temple grounds, comprising both semi-domesticated and feral cats. Based on a stratified random sampling method, 43 temples that host an accessible population of stray cats were randomly selected from 24 districts, with one to three temples per district. A minimum sample size was calculated using the formula $n=\frac{N}{1+N{\left(e\right)}^{2}}$ (Yamane, 1967), assuming a large population size (*N* = 90,000) with a 5% margin of error (*e* = 0.05). To account for potential sample loss or degradation, the target was increased to 500 samples. Samples were collected from 2 to 25 cats per temple based on convenience sampling. Fecal samples were collected individually from the rectum of each cat and sampled cats were temporarily marked to ensure each cat contributed only one sample. Samples were immediately placed into labeled plastic bags, and maintained at 4 °C during transportation to the Department of Parasitology, Faculty of Veterinary Medicine, Kasetsart University.

A structured questionnaire was used to interview the monks or animal caretakers to collect information on age, sex, deworming history, human contact, and sampling zone. Human contact was operationally defined by the cat’s habitual, daily behavioral tolerance toward people. Cats were classified as “yes” if they accepted routine feeding, physical handling, or close proximity to caretakers. Cats were classified as “no” if they exhibited strictly feral behavior, actively avoided human approach, and relied exclusively on scavenging.

This study protocol was approved by the Kasetsart University Institutional Animal Care and Use Committee under the Ethical Review Board of the Office of the National Research Council of Thailand (Approval No. ACKU60-VET-006). Informed consent was obtained from the monks or animal caretakers before sample collection.

### 2.2 DNA extraction

Genomic DNA was extracted from approximately 200 mg of each fecal sample using the E.Z.N.A.^®^ Stool DNA Kit (Omega Bio-tek Inc., Norcross, GA, USA) according to the manufacturer’s instructions and eluted in 100 µL of elution buffer. DNA concentration and purity were measured using a BioSpectrometer (Eppendorf AG, Hamburg, Germany) and stored at -20 °C until further analysis.

### 2.3 Molecular detection

A nested PCR assay targeting the small subunit ribosomal RNA (SSU rRNA) locus was utilized for the initial screening of *G. duodenalis* (Hopkins et al., 1997; Read et al., 2002). Positive samples were subsequently characterized by amplifying the glutamate dehydrogenase (*gdh*) (Cacciò et al., 2008) and triosephosphate isomerase (*tpi*) loci (Sulaiman et al., 2003) to identify specific assemblages and sub-assemblages. All primer sequences and thermocycling conditions are detailed in **Table 1**.

**Table 1:** Oligonucleotide primers and thermocycling conditions used for the molecular detection and genotyping of *G. duodenalis*.

Primer name	Primer sequences (5’-3’)	Cycling condition	Size (bp)	Reference
SSU rRNA		94 °C/3 min		
RH11	CATCCGGTCGATCCTGCC	(94 °C/45 s	292	(Hopkins et al., 1997)
RH4	AGTCGAACCCTGATTCTCCGCCAGG	54 °C/45 s		
GiarF	GACGCTCTCCCCAAGGAC	72 °C/1 min) ×35	174	(Read et al., 2002)
GiarR	CTGCGTCACGCTGCTCG	72 °C/3 min		
*gdh*		94 °C/3 min		
Gdh1	TTCCGTRTYCAGTACAACTC	(94 °C/30 s	754	(Cacciò et al., 2008)
Gdh2	ACCTCGTTCTGRGTGGCGCA	50 °C/30 s		
Gdh3	ATGACYGAGCTYCAGAGGCACGT	72 °C/1 min) ×35	530	
Gdh4	GTGGCGCARGGCATGATGCA	72 °C/7 min		
*tpi*		95 °C/5 min		
TPI-F3	AATAAATIATGCCTGCTCGTCG	(94 °C/45 s	605	(Sulaiman et al., 2003)
TPI-R3	ATGGACITCCTCTGCCTGCTC	50 °C/45 s		
TPI-F4	CCCTTCATCGGIGGTAACTTCAA	72 °C/1 min) ×35	530	
TPI-R2	GTGGCCACCACICCCGTGCC	72 °C/10 min		

PCR amplifications were performed in a final volume of 25 µL. The reaction mixture for the SSU rRNA assay consisted of 16.8 µL of nuclease-free water, 2.5 µL of 10× PCR buffer, 0.75 µL of MgCl_2_ (50 mM), 0.5 µL of dNTPs (10 mM), 1.0 µL each of forward and reverse primers (10 µM), 0.2 µL of i-Taq™ DNA Polymerase (5 U/µL; iNtRON Biotechnology, Seongnam, South Korea), 1.25 µL of DMSO, and 1.0 µL of template DNA (genomic DNA for the primary round, or the primary amplicon for the secondary round). For the *gdh* and *tpi* assays, the reaction mixture was identical except that DMSO was not included, and the volume of nuclease-free water was adjusted to 18.05 µL. A confirmed *G. duodenalis*-positive DNA sample was included as a positive control, and nuclease-free water was included as a negative control in each PCR run. The secondary PCR products were visualized by electrophoresis on a 1.5% agarose gel.

### 2.4 Sequencing and phylogenetic analysis

The positive amplicons were purified using the QIAquick PCR Purification Kit (Qiagen, Hilden, Germany) and submitted to AITbiotech (AITbiotech Pte. Ltd., Singapore) for sequencing. Chromatograms were visually inspected, and consensus sequences were generated using SnapGene^®^ Viewer version 8.2.2 (Dotmatics, Boston, MA, USA). Sequences were compared with reference sequences available in the GenBank database using the Basic Local Alignment Search Tool (BLAST; https://blast.ncbi.nlm.nih.gov/Blast.cgi). Multiple sequence alignments and phylogenetic analyses were performed using MEGA 12 (Kumar et al., 2024). A Maximum Likelihood tree was constructed based on the Tamura 3-parameter substitution model with 1,000 bootstrap replicates.

### 2.5 Statistical analysis

Data were analyzed using IBM SPSS Statistics version 27 (IBM Corp., Armonk, NY, USA). Prevalence was estimated with 95% confidence intervals (CIs) using the Wilson score method. To evaluate factors associated with PCR positivity, univariable analysis was performed using Pearson’s Chi-square or Fisher’s exact tests. Variables with *P* < 0.25 in the univariable analysis were entered into a multivariable logistic regression model. Model fit was assessed using the Hosmer-Lemeshow goodness-of-fit test. Cluster-adjusted analyses were attempted but failed to converge due to sparse data. Standard multivariable logistic regression was retained. Final results are reported as adjusted odds ratios (aOR) with 95% CIs. Associations were considered statistically significant at *P* < 0.05.

## 3 Results

### 3.1 Prevalence of *G. duodenalis* in stray cats

Based on nested PCR amplification of the SSU rRNA gene, the overall prevalence of *G. duodenalis* in the sampled stray cat population was 10.8% (54/500; 95% CI: 8.4–13.8%). The parasite was detected in 12 of the 24 surveyed districts (50.0%) and in 19 of the 43 sampled temples (44.2%). The highest prevalence was observed in Bang Sue district at 30.0% (3/10; 95% CI: 10.8–60.3%), followed by Khlong Sam Wa at 27.6% (8/29; 95% CI: 14.7–45.7%) and Sai Mai at 27.3% (9/33; 95% CI: 15.1–44.2%) (**Table 2**, **Figure 1**).

**Table 2:** Prevalence of *G. duodenalis* infection in temple stray cats stratified by surveyed districts in Bangkok, Thailand.

District	No. temples examined	No. positive temples	No. cats examined	No. cats positive	Prevalence % (95% CI)
Bang Sue	2	2	10	3	30.0 (10.8–60.3)
Khlong Sam Wa	2	2	29	8	27.6 (14.7–45.7)
Sai Mai	3	2	33	9	27.3 (15.1–44.2)
Samphanthawong	2	2	23	6	26.1 (12.5–46.5)
Min Buri	3	3	35	8	22.9 (12.1–39.0)
Bang Kho Laem	2	1	21	4	19.0 (7.7–40.0)
Dusit	2	1	24	4	16.7 (6.7–35.9)
Lat Krabang	3	2	30	5	16.7 (7.3–33.6)
Taling Chan	2	1	19	3	15.8 (5.5–37.6)
Bangkok Noi	1	1	18	2	11.1 (3.1–32.8)
Don Mueang	2	1	24	1	4.2 (0.7–20.2)
Phasi Charoen	2	1	26	1	3.8 (0.7–18.9)
Phra Nakhon	1	0	5	0	0.0 (0.0–43.4)
Bang Khen	1	0	10	0	0.0 (0.0–27.8)
Ratchathewi	1	0	10	0	0.0 (0.0–27.8)
Nong Chok	1	0	14	0	0.0 (0.0–21.5)
Bangkok Yai	1	0	15	0	0.0 (0.0–20.4)
Thon Buri	2	0	16	0	0.0 (0.0–19.4)
Chom Thong	1	0	18	0	0.0 (0.0–17.6)
Lat Phrao	1	0	18	0	0.0 (0.0–17.6)
Yan Nawa	1	0	19	0	0.0 (0.0–16.8)
Chatuchak	1	0	25	0	0.0 (0.0–13.3)
Bang Khun Thian	3	0	26	0	0.0 (0.0–12.9)
Pom Prap Sattru Phai	3	0	32	0	0.0 (0.0–10.7)
Total	43	19	500	54	10.8 (8.4–13.8)

**Figure 1:**
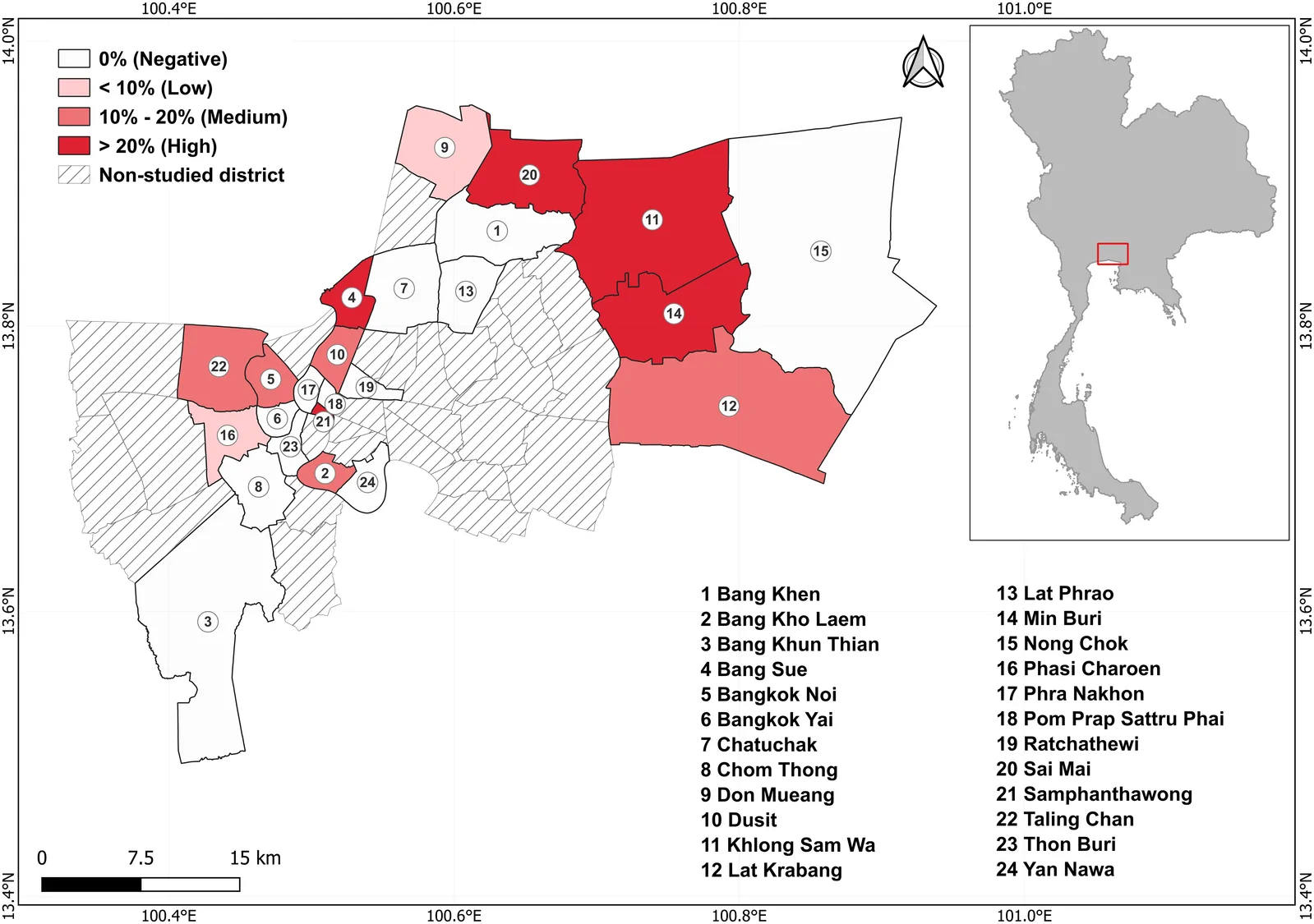
Sampling area and prevalence of *G. duodenalis* infection in temple stray cats across 24 surveyed districts in Bangkok, Thailand. Source: Map generated using QGIS version 3.44.8. Based on data from the Global Administrative Areas (GADM) database.

### 3.2 Sequence analysis and genotyping of *G. duodenalis*

Representative SSU rRNA sequences showed 100% identity to the *G. duodenalis* reference sequence KT595651. Among the 54 positive samples, 7.4% (4/54) and 11.1% (6/54) were successfully genotyped at the *gdh* and *tpi* loci, respectively. Overall, genotyping was achieved for eight isolates: two isolates were characterized at the *gdh* locus only, four isolates at the *tpi* locus only, and multilocus genotyping data were available for two isolates.

Assemblage B (sub-assemblage BIV) was identified in 50.0% (4/8) of the genotyped isolates across the *gdh* (n = 2; 99.37–99.59% identity to OM116001) and *tpi* (n = 2; 99.59–100% identity to ON037509) loci. Assemblage F was detected in 37.5% (3/8) of the isolates, with two isolates at the *gdh* locus (99.79–100% identity to EF507608.1) and three isolates at the *tpi* locus (99.38–99.59% identity to AB569392.1), including the two multilocus-characterized isolates. Assemblage A (sub-assemblage AI) was found in a single isolate (12.5%) at the *tpi* locus (99.58% identity to ON037510).

Complete concordance was observed between BLAST sequence identities and phylogenetic analyses. The sequences generated in this study clustered with their respective reference sequences for sub-assemblages AI and BIV, and assemblage F (**Figures 2**, **3**). All sequences obtained in this study were deposited in the GenBank database under the accession numbers PZ715188–PZ715196 (SSU rRNA), PZ587430–PZ587433 (*gdh*), and PZ587434–PZ587439 (*tpi*).

**Figure 2:**
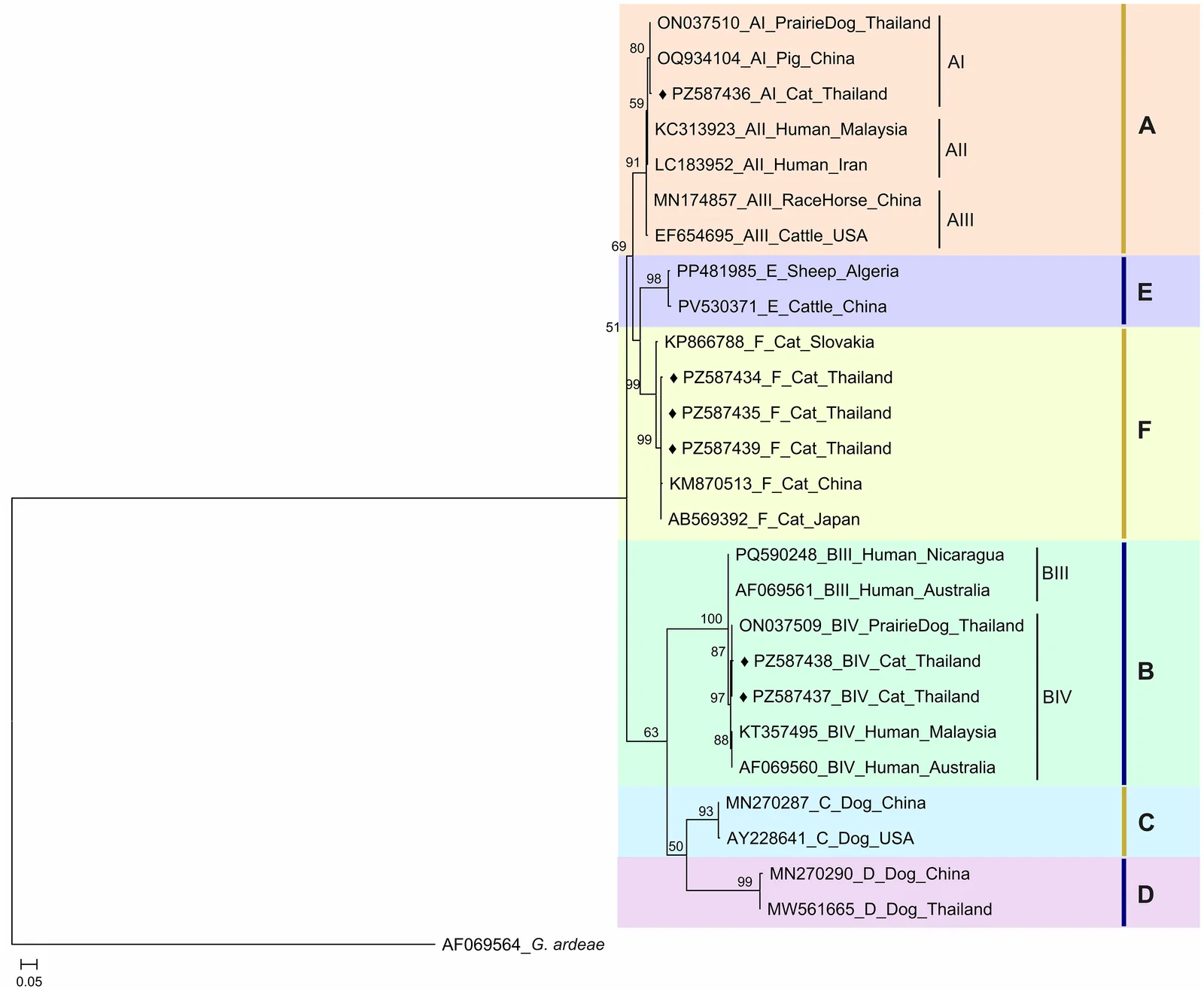
Phylogenetic tree of G. duodenalis at the tpi locus constructed using the maximum likelihood method based on the Tamura 3-parameter model with 1,000 bootstrap replicates. Bootstrap values lower than 50% are not displayed. Filled rhombuses (♦) represent sequences obtained in this study. The labels **(A–F)** denote assemblages **(A–F)**, respectively; AI, AII, and AIII denote sub-assemblages within assemblage A, while BIII and BIV denote sub-assemblages within assemblage B. G. ardeae was used as the outgroup. The scale bar represents 0.05 nucleotide substitutions per site. Source: Generated using MEGA 12. Based on GenBank sequences identified in the article by their accession numbers.

**Figure 3:**
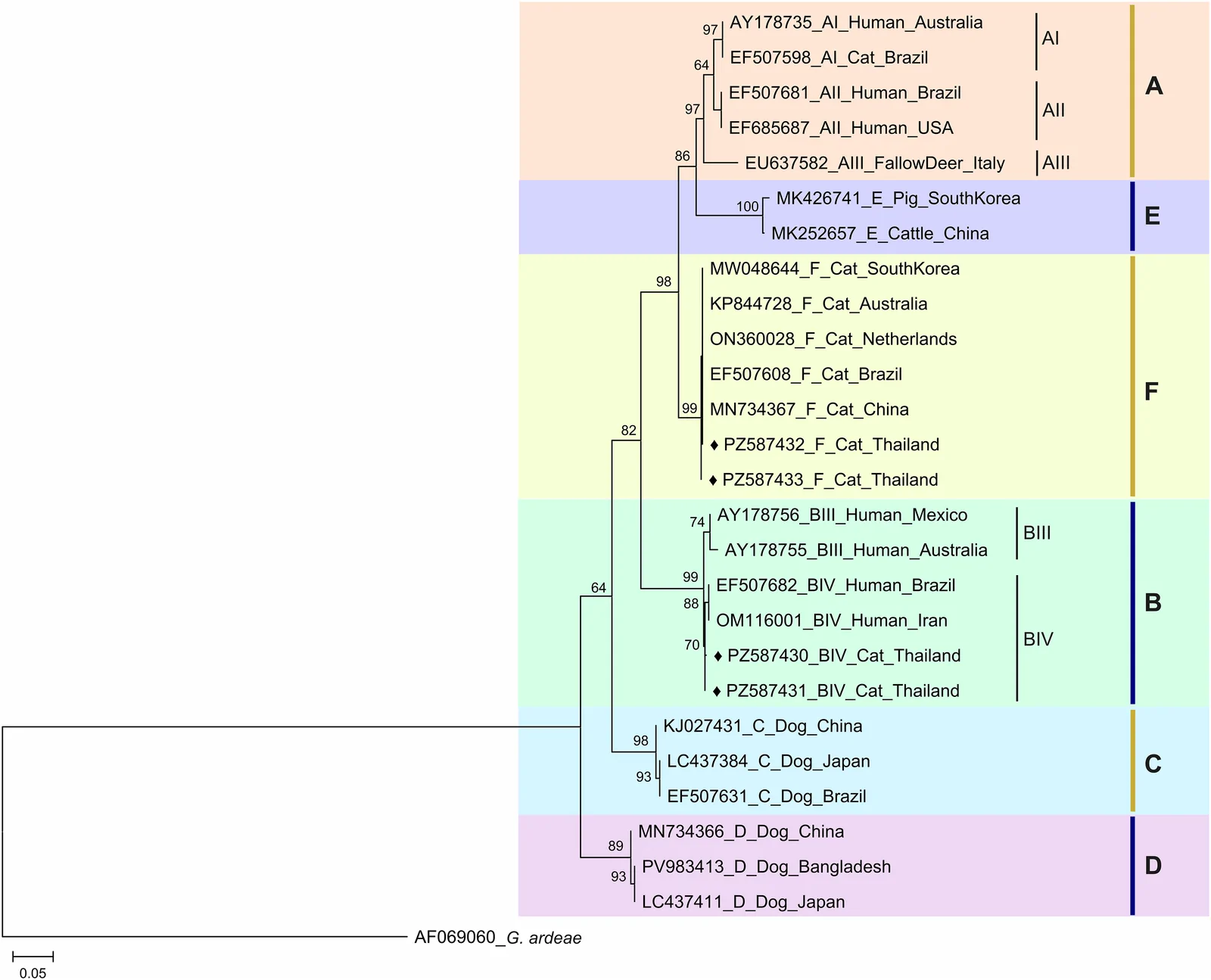
Phylogenetic tree of *G. duodenalis* at the *gdh* locus constructed using the maximum likelihood method based on the Tamura 3-parameter model with 1,000 bootstrap replicates. Bootstrap values lower than 50% are not displayed. Filled rhombuses (♦) represent sequences obtained in this study. The labels **(A–F)** denote assemblages **(A–F)**, respectively; AI, AII, and AIII denote sub-assemblages within assemblage A, while BIII and BIV denote sub-assemblages within assemblage B. G. ardeae was used as the outgroup. The scale bar represents 0.05 nucleotide substitutions per site. Source: Generated using MEGA 12. Based on GenBank sequences identified in the article by their accession numbers.

### 3.3 Factors associated with PCR positivity of *G. duodenalis*

The univariable and multivariable logistic regression analyses of associated factors are detailed in **Table 3**. In the multivariable model, the absence of human contact was identified as an exploratory factor associated with higher odds of PCR positivity (aOR = 8.80; 95% CI: 2.15–35.98; *P* = 0.002). Although sex and geographic zone passed the univariable screening, neither was retained as a significant predictor in the final model (*P* = 0.128 and *P* = 0.068, respectively). Age group and deworming history showed no significant association with *G. duodenalis* PCR positivity.

**Table 3:** Univariable and multivariable logistic regression analyses of factors associated with *G. duodenalis* PCR positivity in temple stray cats.

Factor	No. examined	Positive (%)	Univariable OR (95% CI)	*P*-value	Multivariable aOR (95% CI)	*P*-value
Sex				0.145		0.128
Female	213	28 (13.1%)	Ref		Ref	
Male	287	26 (9.1%)	0.66 (0.37–1.16)		0.64 (0.36–1.14)	
Age group				0.934		–
< 1 year	104	11 (10.6%)	Ref		–	–
≥ 1 year	396	43 (10.9%)	1.03 (0.51–2.08)		–	–
Human contact				0.016*^a^		0.002*
Yes	485	49 (10.1%)	Ref		Ref	
No	15	5 (33.3%)	4.45 (1.46–13.55)		8.80 (2.15–35.98)	
Deworming				0.811		–
Yes	123	14 (11.4%)	Ref		–	–
No	377	40 (10.6%)	0.92 (0.48–1.76)		–	–
Zone				0.203		0.068
Inner city	218	19 (8.7%)	Ref		Ref	
Suburban	177	25 (14.1%)	1.72 (0.91–3.24)	0.092	1.74 (0.92–3.29)	0.086
Urban fringe	105	10 (9.5%)	1.10 (0.49–2.46)	0.812	0.64 (0.23–1.76)	0.384

* *P<* 0.05. ^a^*P* calculated using Fisher’s exact test. Dashes (–) indicate that the variable was not retained in the final multivariable model. OR, odds ratio; aOR, adjusted odds ratio; CI, confidence interval.

## 4 Discussion

*G. duodenalis* is among the most common intestinal protozoa in humans and a diverse range of animals (Adam, 2021; Feng and Xiao, 2011). Globally, it has been reported in cats across 23 countries with a pooled prevalence of 14.5% (Sun et al., 2023). In this study, the prevalence of *G. duodenalis* in stray cats was 10.8% (54/500; 95% CI: 8.4–13.8%). This prevalence is comparable to findings from Australia at 10.1% (Yang et al., 2015), Southern China at 9.8% (Zheng et al., 2015), and Spain at 9.2% (Gil et al., 2017). While lower prevalences have been reported in Iran at 1.5% (Karimi et al., 2023), South Korea at 3.8% (Kwak and Seo, 2020), and parts of China at 2.0–5.8% (Li et al., 2019; Li et al., 2024; Pan et al., 2018), considerably higher infection rates are documented with prevalences reaching 17.9% in Germany (Sommer et al., 2018), 25.0% in Mexico (Veyna-Salazar et al., 2023), 27.9% in Jordan (Mukbel et al., 2024), and 29.9% in Bangladesh (Nahar et al., 2026). This significant variation in prevalence is also documented in Thailand. A study reported a high prevalence of 27.3% (Tangtrongsup et al., 2020), whereas significantly lower infection rates were found at 3.9% (Jitsamai et al., 2021) and 0.3% (Rojekittikhun et al., 2014). Methodological differences may explain these discrepancies. Tangtrongsup et al. (2020) employed a highly sensitive combination of diagnostic techniques, including zinc sulfate centrifugal flotation, immunofluorescent assay (IFA), and molecular analysis. This approach can yield a higher prevalence than studies relying solely on coproantigen ELISA testing (Jitsamai et al., 2021) or conventional microscopic examination (Rojekittikhun et al., 2014). In addition to diagnostic sensitivity, the prevalence of *G. duodenalis* depends on the studied population, environmental conditions, and geographic locations (Enemark et al., 2020; Piekara-Stępińska et al., 2020; Tangtrongsup et al., 2020; Wang et al., 2021).

Determining the *G. duodenalis* assemblage and sub-assemblage is crucial for understanding its zoonotic potential (Guadaño Procesi et al., 2022; Zajaczkowski et al., 2021). While the multi-copy SSU rRNA gene provides superior sensitivity, its highly conserved sequences are primarily utilized for confirming the presence of *G. duodenalis* DNA rather than resolving specific sub-assemblages (Jerez Puebla et al., 2017; Zajaczkowski et al., 2021). Achieving high-resolution genotyping requires highly discriminatory single-copy genes, including *gdh* and *tpi* (Thompson and Ash, 2016; Zajaczkowski et al., 2021). In this study, assemblages B (n = 4), F (n = 3), and A (n = 1) were detected in the surveyed temple stray cats. While the host-adapted assemblage F is predominantly reported in cats globally (Ito et al., 2017; Sun et al., 2023), zoonotic assemblage B emerged as the most frequently detected assemblage among successfully genotyped isolates in this study, followed by assemblage F. This finding aligns with recent epidemiological data from stray cats in Jordan and South Korea (Mukbel et al., 2024; Yun et al., 2023). This apparent similarity should be interpreted cautiously due to potential differences in study populations, sampling strategies, and genotyping methods. The detection of zoonotic assemblages A and B is consistent with findings from various regions, including Germany (Sommer et al., 2018), Japan (Iijima et al., 2018), Spain (Barrera et al., 2026), and Bangladesh (Nahar et al., 2026).

Within assemblages A and B, all successfully genotyped isolates in this study belonged to sub-assemblages AI and BIV, respectively, both of which are considered to have high zoonotic potential (Sprong et al., 2009). Among felines, AI is frequently documented, occasionally followed by AII (Barrera et al., 2026; Iijima et al., 2018; Li et al., 2024; Yang et al., 2015). While the broader assemblage B is frequently reported in dogs and other animals, assigning feline isolates to a specific sub-assemblage is rarely reported, though Barrera et al. (2026) previously identified sub-assemblage BIII in Spanish cats. This limitation is largely attributed to the high genetic polymorphism within assemblage B, which frequently results in double peaks in sequence chromatograms, making definitive identification highly challenging (Zajaczkowski et al., 2021). The detection of sub-assemblages AI and BIV in this study carries particular epidemiological relevance in Thailand, where both sub-assemblages have been found among humans, domestic animals, and wildlife (Kamyingkird et al., 2022; Pipatsatitpong et al., 2018; Siripattanapipong et al., 2011; Tangtrongsup et al., 2019, 2020; Tungtrongchitr et al., 2010). Furthermore, a case of human infection with the feline-adapted assemblage F has been reported in the country (Kotepui et al., 2025). The identification of these assemblages mirrors prior findings in Bangkok temple communities, where concurrent anthroponotic, zoonotic, and dog-specific cycles have been previously proposed (Traub et al., 2009). Although previous epidemiological studies in the region identified cat contact as a risk factor for human giardiasis (Boontanom et al., 2011), the current cross-sectional data cannot determine if the sampled cats actively contribute to zoonotic transmission. As stray cats naturally congregate and share common soil or sand areas for defecation within temple grounds, it is theoretically possible that environmentally robust cysts may accumulate in these communal sites (Barrera et al., 2026; Hatam-Nahavandi et al., 2025). However, genotyping data alone is insufficient to definitively confirm the source of infection or active cross-species transmission. Confirming a zoonotic cycle requires further epidemiological investigation into food, water, and environmental contamination, combined with genetic comparisons of isolates from humans and animals living in the same communities (Cai et al., 2021).

In the surveyed temple stray cat population, the absence of human contact was identified as an exploratory factor associated with *G. duodenalis* PCR positivity. This association may be influenced by socioecological, environmental, and nutritional factors. Stray cats that avoid human contact might exhibit more feral behaviors, potentially relying on scavenging and hunting wild prey. These prey animals, including rodents, which have been reported to harbor assemblages A, B, and F (Li et al., 2023), and reptiles, identified as hosts for zoonotic assemblage B in Thailand (Sutthikornchai et al., 2025), represent plausible sources of exposure. Scavenging activities could theoretically increase the likelihood of exposure to environmental sources that might be contaminated with infective cysts, such as water and soil (Li et al., 2023). In contrast, human interaction may serve as a proxy variable for caregiving. Temple cats that tolerate human contact might be fed by monks and local people, potentially receiving better food and cleaner drinking water. This caregiving could reduce their environmental exposure and reliance on hunting. Furthermore, nutrition can modulate susceptibility to *G. duodenalis* (Gutiérrez and Bartelt, 2024), as inadequate protein intake has been linked to increased susceptibility to giardiasis (Silva et al., 2009). Since these specific scavenging, caregiving, environmental, and nutritional variables were not directly evaluated in this study, they remain untested hypotheses that require further investigation.

Study zones showed no statistically significant association with *G. duodenalis* PCR positivity, consistent with previous studies (Gil et al., 2017; Karimi et al., 2023; Nahar et al., 2026). In this study, *G. duodenalis* was detected across 12 districts and multiple zones. This widespread distribution, combined with the free-roaming behavior of these stray cats, may explain the zone-independent pattern. Deworming history was not associated with PCR positivity, corroborating findings that are widely reported (Nahar et al., 2026; Sun et al., 2023; Yang et al., 2015). The absence of an association can be attributed to the broad timeframe over which treatments were recorded and potential misclassification of the specific anthelmintics administered (Le et al., 2026). In our study, age and sex were not significantly associated with *G. duodenalis* PCR positivity. This demographic-independent pattern has been widely documented for age (Gil et al., 2017; Guadaño Procesi et al., 2022; Li et al., 2019; Nahar et al., 2026; Piekara-Stępińska et al., 2020; Sun et al., 2023) and sex (Gil et al., 2017; Guadaño Procesi et al., 2022; Nahar et al., 2026; Sun et al., 2023; Veyna-Salazar et al., 2023; Yun et al., 2023). Although some studies have reported age and sex as statistical predictors for *G. duodenalis* infection, the literature remains highly inconsistent. While kittens are more susceptible to *G. duodenalis* (Pan et al., 2018; Veyna-Salazar et al., 2023), other research has reported a higher prevalence in elderly cats compared to young cats (Yang et al., 2015). The immunological vulnerability that makes kittens more susceptible to *G. duodenalis* (Kraft et al., 2017) can be offset by the more frequent roaming activities of adult cats for hunting and mating, likely leading to the lack of an age association (Le et al., 2026). Regarding sex, male cats have been reported to have higher infection rates than females (Li et al., 2019), while another study found females to be more susceptible than males (Lider et al., 2025). These differences likely reflect the variability of the studied populations and environments. Given that stray cats have frequent contact with other animals and contaminated environments, this shared ecological exposure can result in similar PCR positivity rates between both sexes.

Several limitations should be considered when interpreting these findings. Since individual cats within the temples were selected via convenience sampling, the findings should be generalized only to the sampled temple stray cats rather than to the wider stray cat population of Bangkok. The reliance on a single cross-sectional sampling may have underestimated the true prevalence of *G. duodenalis* due to the intermittent shedding of cysts. Sparse data prevented the convergence of cluster-adjusted models, meaning potential temple-level clustering could not be statistically controlled. Additionally, the exploratory association identified between PCR positivity and the absence of human contact was based on a highly imbalanced subgroup of only 15 cats; the resulting wide confidence intervals necessitate cautious interpretation. The relatively low genotyping success rate for the *tpi* and *gdh* loci in this study introduces additional diagnostic uncertainty. Such amplification challenges are well-recognized methodological constraints when amplifying single-copy *G. duodenalis* genes directly from fecal samples (Barrera et al., 2026), and were likely compounded by prolonged DNA storage in this study. Because of these constraints, although no mixed assemblage infections were detected in the present study, their presence cannot be entirely ruled out. Addressing these diagnostic challenges is critical in Thailand, where molecular techniques are underutilized, restricting the generation of comprehensive epidemiological data regarding *G. duodenalis* assemblage distribution, which is essential for fully understanding host specificity and transmission sources (Kotepui et al., 2025).

To our knowledge, this is the first molecular epidemiological study of *G. duodenalis* in temple stray cats in Thailand, providing baseline data on its prevalence and the detection of both host-adapted and zoonotic assemblages. Exploratory analysis identified the absence of human contact as a factor associated with PCR positivity. Future studies incorporating paired sampling across human, animal, and environmental interfaces are necessary to elucidate actual transmission pathways and to evaluate potential One Health interventions.

## Data Availability

Publicly available datasets were analyzed in this study. This data can be found here: NCBI GenBank, accessions PZ715188–PZ715196 (SSU rRNA), PZ587430–PZ587433 (gdh), and PZ587434–PZ587439 (tpi).

## References

[B1] Adam R. D. (2021). *Giardia duodenalis*: biology and pathogenesis. Clin. Microbiol. Rev. 34, e00024-19. doi: 10.1128/CMR.00024-19 34378955 PMC8404698

[B2] Ballweber L. R. Xiao L. Bowman D. D. Kahn G. Cama V. A. (2010). Giardiasis in dogs and cats: update on epidemiology and public health significance. Trends Parasitol. 26, 180–189. doi: 10.1016/j.pt.2010.02.005 20202906

[B3] Barrera J. P. Montoya A. de Lucio A. Köster P. C. Bailo B. Carmena D. . (2026). Molecular characterization and zoonotic potential of *Giardia* and *Cryptosporidium* infections in dogs and cats in central Spain. Food Waterborne Parasitol. 44, e00351. doi: 10.1016/j.fawpar.2026.e00351 42381722 PMC13315442

[B4] Boontanom P. Mungthin M. Tan-ariya P. Naaglor T. Leelayoova S. (2011). Epidemiology of giardiasis and genotypic characterization of *Giardia duodenalis* in preschool children of a rural community, central Thailand. Trop. Biomed. 28, 32–39. 21602766

[B5] Cacciò S. M. Beck R. Lalle M. Marinculic A. Pozio E. (2008). Multilocus genotyping of *Giardia duodenalis* reveals striking differences between assemblages A and B. Int. J. Parasitol. 38, 1523–1531. doi: 10.1016/j.ijpara.2008.04.008 18571176

[B6] Cai W. Ryan U. Xiao L. Feng Y. (2021). Zoonotic giardiasis: an update. Parasitol. Res. 120, 4199–4218. doi: 10.1007/s00436-021-07325-2 34623485

[B7] Certad G. Viscogliosi E. Chabé M. Cacciò S. M. (2017). Pathogenic mechanisms of *cryptosporidium* and *giardia*. Trends Parasitol. 33, 561–576. doi: 10.1016/j.pt.2017.02.006 28336217

[B8] Dixon B. R. (2021). *Giardia duodenalis* in humans and animals – transmission and disease. Res. Vet. Sci. 135, 283–289. doi: 10.1016/j.rvsc.2020.09.034 33066992

[B9] Enemark H. L. Starostka T. P. Larsen B. Takeuchi-Storm N. Thamsborg S. M. (2020). *Giardia* and *Cryptosporidium* infections in Danish cats: risk factors and zoonotic potential. Parasitol. Res. 119, 2275–2286. doi: 10.1007/s00436-020-06715-2 32451716

[B10] Faria C. P. Zanini G. M. Dias G. S. da Silva S. Sousa M. C. (2017). New multilocus genotypes of *Giardia lamblia* human isolates. Infect. Genet. Evol. 54, 128–137. doi: 10.1016/j.meegid.2017.06.028 28669825

[B11] Feng Y. Xiao L. (2011). Zoonotic potential and molecular epidemiology of *Giardia* species and giardiasis. Clin. Microbiol. Rev. 24, 110–140. doi: 10.1128/CMR.00033-10 21233509 PMC3021202

[B12] Gil H. Cano L. de Lucio A. Bailo B. de Mingo M. H. Cardona G. A. . (2017). Detection and molecular diversity of *Giardia duodenalis* and *Cryptosporidium* spp. in sheltered dogs and cats in Northern Spain. Infect. Genet. Evol. 50, 62–69. doi: 10.1016/j.meegid.2017.02.013 28219812

[B13] Guadaño Procesi I. Carnio A. Berrilli F. Montalbano Di Filippo M. Scarito A. Amoruso C. . (2022). *Giardia duodenalis* in colony stray cats from Italy. Zoonoses Public Health 69, 46–54. doi: 10.1111/zph.12894 34492169 PMC9290339

[B14] Gutiérrez L. Bartelt L. (2024). Current understanding of *Giardia lamblia* and pathogenesis of stunting and cognitive deficits in children from low- and middle-income countries. Curr. Trop. Med. Rep. 11, 28–39. doi: 10.1007/s40475-024-00314-2 38993355 PMC11238937

[B15] Hatam-Nahavandi K. Ahmadpour E. Badri M. Eslahi A. V. Anvari D. Carmena D. . (2025). Global prevalence of *Giardia* infection in nonhuman mammalian hosts: a systematic review and meta-analysis of five million animals. PloS Negl.Trop. Dis. 19, e0013021. doi: 10.1371/journal.pntd.0013021 40273200 PMC12052165

[B16] Hernández P. C. Morales L. Chaparro-Olaya J. Sarmiento D. Jaramillo J. F. Ordoñez G. A. . (2019). Intestinal parasitic infections and associated factors in children of three rural schools in Colombia. A cross-sectional study. PloS One 14, e0218681. doi: 10.1371/journal.pone.0218681 31291262 PMC6619675

[B17] Hopkins R. M. Meloni B. P. Groth D. M. Wetherall J. D. Reynoldson J. A. Thompson R. C. A. (1997). Ribosomal RNA sequencing reveals differences between the genotypes of *Giardia* isolates recovered from humans and dogs living in the same locality. J. Parasitol. 83, 44–51. doi: 10.2307/3284315 9057695

[B18] Iijima Y. Itoh N. Ito Y. Kimura Y. (2018). Multilocus genotyping of *Giardia duodenalis* isolates from household cats and pet shop kittens. Vet. Parasitol. 259, 44–48. doi: 10.1016/j.vetpar.2018.06.022 30056982

[B19] Inpankaew T. Jiyipong T. Thadtapong N. Kengradomkij C. Pinyopanuwat N. Chimnoi W. . (2015). Prevalence and genotype of *Giardia duodenalis* in dairy cattle from northern and northeastern part of Thailand. Acta Parasitol. 60, 459–461. doi: 10.1515/ap-2015-0063 26204183

[B20] Inpankaew T. Traub R. J. Thompson R. C. Sukthana Y. (2007). Canine parasitic zoonoses in Bangkok temples. Southeast. Asian J. Trop. Med. Public Health 38, 247–255. 17539273

[B21] Ito Y. Iijima Y. Itoh N. Kimura Y. (2017). Multilocus genotyping of *Giardia duodenalis* isolates from breeding cattery cats in Japan. J. Feline Med. Surg. Open Rep. 3, 2055116917745237. doi: 10.1177/2055116917745237 29270308 PMC5731622

[B22] Iwashita H. Sugamoto T. Takemura T. Tokizawa A. Vu T. D. Nguyen T. H. . (2020). Molecular epidemiology of *Giardia* spp. in northern Vietnam: potential transmission between animals and humans. Parasite Epidemiol. Control 12, e00193. doi: 10.1016/j.parepi.2020.e00193 33490658 PMC7806796

[B23] Jerez Puebla L. E. Núñez F. A. Santos L. P. Rivero L. R. Silva I. M. Valdés L. A. . (2017). Molecular analysis of *Giardia duodenalis* isolates from symptomatic and asymptomatic children from La Habana, Cuba. Parasite Epidemiol. Control 2, 105–113. doi: 10.1016/j.parepi.2017.05.003 29774288 PMC5952668

[B24] Jitsamai W. Khrutkham N. Hunprasit V. Chandrashekar R. Bowman D. Sukhumavasi W. (2021). Prevalence of endoparasitic and viral infections in client-owned cats in metropolitan Bangkok, Thailand, and the risk factors associated with feline hookworm infections. Vet. Parasitol. Reg. Stud. Rep. 25, 100584. doi: 10.1016/j.vprsr.2021.100584 34474779

[B25] Kamyingkird K. Phoosangwalthong P. Klinkaew N. Leelanupat A. Kengradomkij C. Chimnoi W. . (2022). Detection of *Giardia duodenalis* zoonotic assemblages AI and BIV in pet prairie dogs (*Cynomys ludovicianus*) in Bangkok, Thailand. Animals 12, 1949. doi: 10.3390/ani12151949 35953937 PMC9367409

[B26] Karimi P. Shafaghi-Sisi S. Meamar A. R. Razmjou E. (2023). Molecular identification of *Cryptosporidium*, *Giardia*, and *Blastocystis* from stray and household cats and cat owners in Tehran, Iran. Sci. Rep. 13, 1554. doi: 10.1038/s41598-023-28768-w 36707690 PMC9883249

[B27] Khine N. O. Chimnoi W. Kamyingkird K. Kengradomkij C. Saetiew N. Simking P. . (2021). Molecular detection of *Giardia duodenalis* and *Cryptosporidium* spp. from stray dogs residing in monasteries in Bangkok, Thailand. Parasitol. Int. 83, 102337. doi: 10.1016/j.parint.2021.102337 33771679

[B28] Khurajog B. Masakul A. Inpankaew T. Kamyingkird K. Wongpanit K. Jittapalapong S. (2014). Prevalence of *Giardia duodenalis* and factors associated with its infection in water buffaloes in Northeast Thailand. J. Trop. Med. Parasitol. 37, 35–42.

[B29] Kitvatanachai S. Jantrapanukorn B. Supcharoengoon U. Atasilp C. (2021). Enteropathogenic bacterial and intestinal parasitic infections among asymptomatic food handlers in Rangsit University canteens, central Thailand. J. Parasitol. Res. 2021, 5565014. doi: 10.1155/2021/5565014 33936804 PMC8055383

[B30] Kotepui M. Popruk S. Kotepui K. U. Masangkay F. R. Wangdi K. Mahittikorn A. (2025). Prevalence and genotyping of *Giardia duodenalis* infections in humans in Thailand: a systematic review and meta-analysis. BMC Infect. Dis. 26, 131. doi: 10.1186/s12879-025-12372-6 41422070 PMC12831254

[B31] Kraft M. R. Klotz C. Bücker R. Schulzke J. D. Aebischer T. (2017). *Giardia*’s epithelial cell interaction *in vitro*: mimicking asymptomatic infection? Front. Cell. Infect. Microbiol. 7, 421. doi: 10.3389/fcimb.2017.00421 29018775 PMC5622925

[B32] Kumar S. Stecher G. Suleski M. Sanderford M. Sharma S. Tamura K. (2024). MEGA12: molecular evolutionary genetic analysis version 12 for adaptive and green computing. Mol. Biol. Evol. 41, 1–9. doi: 10.1093/molbev/msae263 39708372 PMC11683415

[B33] Kurnosova O. P. Panova O. A. Arisov M. V. (2024). Prevalence of *Giardia duodenalis* in dogs and cats: age-related predisposition, symptomatic, and asymptomatic cyst shedding. Vet. World 17, 379–383. doi: 10.14202/vetworld.2024.379-383 38595671 PMC11000481

[B34] Kwak D. Seo M. G. (2020). Genetic analysis of zoonotic gastrointestinal protozoa and microsporidia in shelter cats in South Korea. Pathogens 9, 894. doi: 10.3390/pathogens9110894 33121067 PMC7693795

[B35] Le V. T. T. Hoang M. T. T. Ngo V. P. Ng-Nguyen D. Inpankaew T. (2026). Molecular detection of *Giardia duodenalis* and the absence of *Cryptosporidium* spp. in owned dogs from central Vietnam. Front. Parasitol. 5, 1860216. doi: 10.3389/fpara.2026.1860216 42311390 PMC13269053

[B36] Li J. Dan X. Zhu K. Li N. Guo Y. Zheng Z. . (2019). Genetic characterization of *Cryptosporidium* spp. and *Giardia duodenalis* in dogs and cats in Guangdong, China. Parasitol. Vectors 12, 571. doi: 10.1186/s13071-019-3822-z 31783765 PMC6884805

[B37] Li J. Qin H. Li X. Zhang L. (2023). Role of rodents in the zoonotic transmission of giardiasis. One Health 16, 100500. doi: 10.1016/j.onehlt.2023.100500 36844973 PMC9947413

[B38] Li L. Sui Y. Li X. Song P. Chen G. Liu H. . (2024). Molecular characterization of *Cryptosporidium* spp. and *Giardia duodenalis* in pet cats in Henan Province, central China. Acta Trop. 254, 107188. doi: 10.1016/j.actatropica.2024.107188 38531428

[B39] Lider L. A. Kiyan V. Seitkamzina D. M. Ussenbayev A. Akmambaeva B. E. Uakhit R. S. . (2025). Prevalence, risk factors, and zoonotic implications of gastrointestinal parasites in urban cats in Kazakhstan: a cross-sectional multicity study. Vet. World 18, 1748–1758. doi: 10.14202/vetworld.2025.1748-1758 40689166 PMC12269952

[B40] Mukbel R. Hammad H. Enemark H. Alsabi R. Al-Sabi M. (2024). Molecular characterization of *Giardia duodenalis*, *Cryptosporidium* spp. and *Entamoeba* spp. infecting domestic and feral/stray cats in Jordan. Parasitol. Res. 123, 351. doi: 10.1007/s00436-024-08358-z 39404859

[B41] Nahar A. Hasan M. F. Harun A. B. Bayazid A. A. Sultana T. Rehena J. . (2026). Molecular detection and zoonotic potential of *Cryptosporidium* spp. and *Giardia duodenalis* in cats and dogs from metropolitan areas of Bangladesh. Food Waterborne Parasitol. 42, e00314. doi: 10.1016/j.fawpar.2025.e00314 41552711 PMC12809397

[B42] Pan W. Wang M. Abdullahi A. Y. Fu Y. Yan X. Yang F. . (2018). Prevalence and genotypes of *Giardia lamblia* from stray dogs and cats in Guangdong, China. Vet. Parasitol. Reg. Stud. Rep. 13, 30–34. doi: 10.1016/j.vprsr.2018.03.012 31014884

[B43] Phoosangwalthong P. Kamyingkird K. Kengradomkij C. Chimnoi W. Odermatt P. Inpankaew T. (2023). Molecular detection and genetic characterization of zoonotic hookworm in semi-domesticated cats residing in monasteries in Bangkok, Thailand. Trop. Med. Infect. Dis. 8, 122. doi: 10.3390/tropicalmed8020122 36828538 PMC9960371

[B44] Phoosangwalthong P. Luong N. H. Wongwigkan J. Kamyingkird K. Phasuk J. Pattanatanang K. . (2022). *Toxocara canis* and *Toxocara cati* in stray dogs and cats in Bangkok, Thailand: molecular prevalence and risk factors. Parasitologia 2, 88–94. doi: 10.3390/parasitologia202000930654563

[B45] Piekara-Stępińska A. Piekarska J. Gorczykowski M. Bania J. (2020). Genotypes of *Giardia duodenalis* in household dogs and cats from Poland. Acta Parasitol. 66, 428–435. doi: 10.1007/s11686-020-00292-1 33040220 PMC8166709

[B46] Pipatsatitpong D. Aunpad R. Siripattanapipong S. Mungthin M. Leelayoova S. (2018). Prevalence and genotype of *Giardia duodenalis* of asymptomatic individual in a child care center, Bangkok, Thailand. Sci. Technol. Asia 23, 22–27. doi: 10.14456/scitechasia.2018.20

[B47] Rahimi H. M. Javanmard E. Taghipour A. Haghighi A. Mirjalali H. (2023). Multigene typing of *Giardia duodenalis* isolated from tuberculosis and non-tuberculosis subjects. PloS One 18, e0283515. doi: 10.1371/journal.pone.0283515 36952535 PMC10035859

[B48] Read C. Walters J. Robertson I. D. Thompson R. C. A. (2002). Correlation between genotype of *Giardia duodenalis* and diarrhoea. Int. J. Parasitol. 32, 229–231. doi: 10.1016/s0020-7519(01)00340-x 11812501

[B49] Rojekittikhun W. Chaisiri K. Mahittikorn A. Pubampen S. Sa-nguankiat S. Kusolsuk T. . (2014). Gastrointestinal parasites of dogs and cats in a refuge in Nakhon Nayok, Thailand. Southeast. Asian J. Trop. Med. Public Health 45, 31–39. 24964651

[B50] Ryan U. Cacciò S. M. (2013). Zoonotic potential of *giardia*. Int. J. Parasitol. 43, 943–956. doi: 10.1016/j.ijpara.2013.06.001 23856595

[B51] Ryan U. M. Feng Y. Fayer R. Xiao L. (2021). Taxonomy and molecular epidemiology of *Cryptosporidium* and *Giardia* – a 50-year perspective, (1971–2021). Int. J. Parasitol. 51, 1099–1119. doi: 10.1016/j.ijpara.2021.08.007 34715087

[B52] Saleh M. N. Lindsay D. S. Leib M. S. Zajac A. M. (2019). *Giardia duodenalis* assemblages in cats from Virginia, USA. Vet. Parasitol. Reg. Stud. Rep. 15, 100257. doi: 10.1016/j.vprsr.2018.100257 30929934

[B53] Silva R. R. da Silva C. A. M. de Jesus Pereira C. A. de Carvalho Nicolato R. L. Negrão-Corrêa D. Lamounier J. A. . (2009). Association between nutritional status, environmental and socio-economic factors and *Giardia lamblia* infections among children aged 6–71 months in Brazil. Trans. R. Soc Trop. Med. Hyg. 103, 512–519. doi: 10.1016/j.trstmh.2008.10.019 19054535

[B54] Siripattanapipong S. Leelayoova S. Mungthin M. Thompson R. A. Boontanom P. Saksirisampant W. . (2011). Clonal diversity of the glutamate dehydrogenase gene in *Giardia duodenalis* from Thai isolates: evidence of genetic exchange or mixed infections? BMC Microbiol. 11, 206. doi: 10.1186/1471-2180-11-206 21933419 PMC3191338

[B55] Sommer M. F. Rupp P. Pietsch M. Kaspar A. Beelitz P. (2018). *Giardia* in a selected population of dogs and cats in Germany – diagnostics, coinfections and assemblages. Vet. Parasitol. 249, 49–56. doi: 10.1016/j.vetpar.2017.11.006 29279086

[B56] Sprong H. Cacciò S. M. Van Der Giessen J. W. B. (2009). Identification of zoonotic genotypes of *Giardia duodenalis*. PloS Negl.Trop. Dis. 3, e558. doi: 10.1371/journal.pntd.0000558 19956662 PMC2777335

[B57] Sricharern W. Inpankaew T. Keawmongkol S. Supanam J. Stich R. W. Jittapalapong S. (2016). Molecular detection and prevalence of *Giardia duodenalis* and *Cryptosporidium* spp. among long-tailed macaques (*Macaca fascicularis*) in Thailand. Infect. Genet. Evol. 40, 310–314. doi: 10.1016/j.meegid.2016.02.004 26892616

[B58] Sulaiman I. M. Fayer R. Bern C. Gilman R. H. Trout J. M. Schantz P. M. . (2003). Triosephosphate isomerase gene characterization and potential zoonotic transmission of *Giardia duodenalis*. Emerg. Infect. Dis. 9, 1444–1452. doi: 10.3201/eid0911.030084 14718089 PMC3035538

[B59] Sun J. Qin Z. Fu Y. Qin H. Sun M. Dong H. . (2023). Assessment of potential zoonotic transmission of *Giardia duodenalis* from dogs and cats. One Health 17, 100651. doi: 10.1016/j.onehlt.2023.100651 38116451 PMC10728314

[B60] Sutthikornchai C. Pintong A. R. Udonsom R. Khulmanee T. Chiabchalard R. Mahittikorn A. . (2025). Detection of *Cryptosporidium* and *Giardia duodenalis* in reptiles in Thailand. Vet. Med. Sci. 12, e70704. doi: 10.1002/vms3.70704 41317138 PMC12664283

[B61] Tangtrongsup S. Scorza A. V. Reif J. S. Ballweber L. R. Lappin M. R. Salman M. D. (2017). Prevalence and multilocus genotyping analysis of *Cryptosporidium* and *Giardia* isolates from dogs in Chiang Mai, Thailand. Vet. Sci. 4, 26. doi: 10.3390/vetsci4020026 29056685 PMC5606608

[B62] Tangtrongsup S. Scorza A. V. Reif J. S. Ballweber L. R. Lappin M. R. Salman M. D. (2020). Seasonal distributions and other risk factors for *Giardia duodenalis* and *Cryptosporidium* spp. infections in dogs and cats in Chiang Mai, Thailand. Prev. Vet. Med. 174, 104820. doi: 10.1016/j.prevetmed.2019.104820 31739222

[B63] Tangtrongsup S. Sripakdee D. Malaivijitnond S. Angkuratipakorn R. Lappin M. (2019). Intestinal parasites and the occurrence of zoonotic *Giardia duodenalis* genotype in captive gibbons at Krabokkoo Wildlife Breeding Center, Thailand. Front. Vet. Sci. 6, 110. doi: 10.3389/fvets.2019.00110 31106211 PMC6499157

[B64] Thompson R. C. A. Ash A. (2016). Molecular epidemiology of *Giardia* and *Cryptosporidium* infections. Infect. Genet. Evol. 40, 315–323. doi: 10.1016/j.meegid.2015.09.028 26458528

[B65] Traub R. J. Inpankaew T. Reid S. A. Sutthikornchai C. Sukthana Y. Robertson I. D. . (2009). Transmission cycles of *Giardia duodenalis* in dogs and humans in Temple communities in Bangkok—a critical evaluation of its prevalence using three diagnostic tests in the field in the absence of a gold standard. Acta Trop. 111, 125–132. doi: 10.1016/j.actatropica.2009.03.006 19524080

[B66] Tungtrongchitr A. Sookrung N. Indrawattana N. Kwangsi S. Ongrotchanakun J. Chaicumpa W. (2010). *Giardia intestinalis* in Thailand: identification of genotypes. J. Health Popul. Nutr. 28, 42–52. doi: 10.3329/jhpn.v28i1.4522 20214085 PMC2975845

[B67] Veyna-Salazar N. P. Cantó-Alarcón G. J. Olvera-Ramírez A. M. Ruiz-López F. J. Bernal-Reynaga R. Bárcenas-Reyes I. . (2023). Occurrence of *Giardia duodenalis* in cats from Queretaro and the risk to public health. Animals 13, 1098. doi: 10.3390/ani13061098 36978639 PMC10044519

[B68] Wang Y. G. Zou Y. Yu Z. Z. Chen D. Gui B. Z. Yang J. F. . (2021). Molecular investigation of zoonotic intestinal protozoa in pet dogs and cats in Yunnan Province, southwestern China. Pathogens 10, 1107. doi: 10.3390/pathogens10091107 34578141 PMC8466027

[B69] Yamane T. (1967). Statistics: An Introductory Analysis. 2nd Edn (New York, NY: Harper & Row).

[B70] Yang R. Ying J. L. J. Monis P. Ryan U. (2015). Molecular characterisation of *Cryptosporidium* and *Giardia* in cats (*Felis catus*) in Western Australia. Exp. Parasitol. 155, 13–18. doi: 10.1016/j.exppara.2015.05.001 25959691

[B71] Yun C. S. Moon B. Y. Lee K. Kang S. M. Ku B. K. Hwang M. H. (2023). The detection and phylogenetic characterization of *Cryptosporidium*, *Cystoisospora*, and *Giardia duodenalis* of cats in South Korea. Front. Cell. Infect. Microbiol. 13, 1296118. doi: 10.3389/fcimb.2023.1296118 38029266 PMC10663360

[B72] Zajaczkowski P. Lee R. Fletcher-Lartey S. M. Alexander K. Mahimbo A. Stark D. . (2021). The controversies surrounding *Giardia intestinalis* assemblages A and B. Curr. Res. Parasitol. Vector-Borne Dis. 1, 100055. doi: 10.1016/j.crpvbd.2021.100055 35284870 PMC8906113

[B73] Zheng G. Hu W. Liu Y. Luo Q. Tan L. Li G. (2015). Occurrence and molecular identification of *Giardia duodenalis* from stray cats in Guangzhou, southern China. Korean J. Parasitol. 53, 119–123. doi: 10.3347/kjp.2015.53.1.119 25748719 PMC4384803

